# Interactions between commensal *Enterococcus faecium* and *Enterococcus lactis* and clinical isolates of *Enterococcus faecium*

**DOI:** 10.1093/femsmc/xtae009

**Published:** 2024-03-09

**Authors:** Theresa Maria Wagner, Anna Kaarina Pöntinen, Carolin Kornelia Fenzel, Daniel Engi, Jessin Janice, Ana C Almeida-Santos, Ana P Tedim, Ana R Freitas, Luísa Peixe, Willem van Schaik, Mona Johannessen, Kristin Hegstad

**Affiliations:** Research group for Host-Microbe Interactions, Department of Medical Biology, Faculty of Health Sciences, UiT The Arctic University of Norway, 9037 Tromsø, Norway; Norwegian National Advisory Unit on Detection of Antimicrobial Resistance, Department of Microbiology and Infection Control, University Hospital of North Norway, 9038 Tromsø, Norway; Department of Biostatistics, Faculty of Medicine, University of Oslo, 0372 Oslo, Norway; Research group for Host-Microbe Interactions, Department of Medical Biology, Faculty of Health Sciences, UiT The Arctic University of Norway, 9037 Tromsø, Norway; Research group for Host-Microbe Interactions, Department of Medical Biology, Faculty of Health Sciences, UiT The Arctic University of Norway, 9037 Tromsø, Norway; Research group for Host-Microbe Interactions, Department of Medical Biology, Faculty of Health Sciences, UiT The Arctic University of Norway, 9037 Tromsø, Norway; Norwegian National Advisory Unit on Detection of Antimicrobial Resistance, Department of Microbiology and Infection Control, University Hospital of North Norway, 9038 Tromsø, Norway; UCIBIO. Departamento de Ciências Biológicas, Laboratório de Microbiologia. Faculdade de Farmácia. Universidade do Porto, 4050-313 Porto, Portugal; Associate Laboratory i4HB, Institute for Health and Bioeconomy, Faculty of Pharmacy, University of Porto, 4050-313 Porto, Portugal; Group for Biomedical Research in Sepsis (BioSepsis), Instituto de Investigación Biomédica de Salamanca, 37007 Salamanca, Spain; Centro de Investigación Biomédica en Red Enfermedades Respiratorias (CiberES CB22/06/00035), 28029 Madrid, Spain; UCIBIO. Departamento de Ciências Biológicas, Laboratório de Microbiologia. Faculdade de Farmácia. Universidade do Porto, 4050-313 Porto, Portugal; Associate Laboratory i4HB, Institute for Health and Bioeconomy, Faculty of Pharmacy, University of Porto, 4050-313 Porto, Portugal; 1H- TOXRUN – One Health Toxicology Research Unit, University Institute of Health Sciences, CESPU, 4584-116 Gandra, Portugal; UCIBIO. Departamento de Ciências Biológicas, Laboratório de Microbiologia. Faculdade de Farmácia. Universidade do Porto, 4050-313 Porto, Portugal; Associate Laboratory i4HB, Institute for Health and Bioeconomy, Faculty of Pharmacy, University of Porto, 4050-313 Porto, Portugal; Institute of Microbiology and Infection, College of Medical and Dental Sciences, University of Birmingham, Birmingham B15 2TT, United Kingdom; Research group for Host-Microbe Interactions, Department of Medical Biology, Faculty of Health Sciences, UiT The Arctic University of Norway, 9037 Tromsø, Norway; Research group for Host-Microbe Interactions, Department of Medical Biology, Faculty of Health Sciences, UiT The Arctic University of Norway, 9037 Tromsø, Norway; Norwegian National Advisory Unit on Detection of Antimicrobial Resistance, Department of Microbiology and Infection Control, University Hospital of North Norway, 9038 Tromsø, Norway

**Keywords:** Enterococcus, nosocomial, commensal, inhibition, bacteriocin

## Abstract

*Enterococcus faecium* (*Efm*) is a versatile pathogen, responsible for multidrug-resistant infections, especially in hospitalized immunocompromised patients. Its population structure has been characterized by diverse clades (A1, A2, and B (reclassified as *E. lactis* (*Ela*)), adapted to different environments, and distinguished by their resistomes and virulomes. These features only partially explain the predominance of clade A1 strains in nosocomial infections. We investigated in vitro interaction of 50 clinical isolates (clade A1 *Efm*) against 75 commensal faecal isolates from healthy humans (25 clade A2 *Efm* and 50 *Ela*). Only 36% of the commensal isolates inhibited clinical isolates, while 76% of the clinical isolates inhibited commensal isolates. The most apparent overall differences in inhibition patterns were presented between clades. The inhibitory activity was mainly mediated by secreted, proteinaceous, heat-stable compounds, likely indicating an involvement of bacteriocins. A custom-made database targeting 76 Bacillota bacteriocins was used to reveal bacteriocins in the genomes. Our systematic screening of the interactions between nosocomial and commensal *Efm* and *Ela* on a large scale suggests that, in a clinical setting, nosocomial strains not only have an advantage over commensal strains due to their possession of AMR genes, virulence factors, and resilience but also inhibit the growth of commensal strains.

## Introduction

*Enterococcus faecium* colonizes the gastrointestinal tract of healthy individuals and animals but has in recent years emerged as a nosocomial pathogen (Arias and Murray 2012). *Enterococcus faecium* infections are an increasing concern in health care, due to its high intrinsic antimicrobial resistance, its capacity to acquire novel resistance genes, and its ability to withstand harsh conditions, including disinfectants (Arias and Murray 2012, Wagenvoort et al. 2015, Pidot et al. 2018).

The clade structure of *E. faecium* has been characterized by a deep phylogenetic split, with clade B dominating in the community (Lebreton et al. 2013), which was recently reclassified as *Enterococcus lactis* (Belloso Daza et al. 2021). *E. faecium* clades are further split into clade A1 and A2, where clade A1 almost exclusively accounts for infections and overlaps with the former clonal complex 17 (CC17) (Leavis et al. 2007, Guzman Prieto et al. 2016). Clade A2 has historically been mainly associated with livestock and domestic animals (Lebreton et al. 2013, Gouliouris et al. 2018), and non-hospital-associated human isolates (Arredondo-Alonso et al. 2020). The clades differ in their accessory and core genome, enabling adaption to different niches (Galloway-Peña et al. 2012, Palmer et al. 2012, van Hal et al. 2021, AL-Rubaye et al. 2023). The accessory genome of clade A1 is enriched in acquired antibiotic resistance determinants (Leavis et al. 2006), genomic islands (van Schaik et al. 2010), specific insertion sequences (Leavis et al. 2007, Werner et al. 2011), and virulence factors (Gao et al. 2018, AL-Rubaye et al. 2023). *Enterococcus lactis* is generally more antibiotic susceptible, while clade *E. faecium* A1 strains are frequently resistant to multiple antibiotics, including ampicillin, due to differences in *pbp5* sequence and its expression (Pietta et al. 2014), and also acquired resistance to vancomycin, aminoglycosides, and linezolid (Willems et al. 2005, AL-Rubaye et al. 2023). Clade A1 strains are predominant in hospitalized patients (Ubeda et al. 2010, Taur et al. 2012) and infection is generally preceded by asymptomatic colonization with antibiotic-resistant strains and within-host evolution (Ubeda et al. 2010, Moradigaravand et al. 2017, Bayjanov et al. 2019). Colonized patients may then contaminate healthcare workers as well as their surroundings, which is accelerated by *E. faecium's* ability to withstand harsh conditions (Wendt et al. 1998, Pidot et al. 2018), in turn leading to outbreaks (de Regt et al. 2008). Additionally, it has been shown that commensal but not clinical *E. faecium* strains are susceptible to group IIA-Secreted phospholipase A_2_ in human serum, although Gram-positive bacteria are considered resistant to killing by serum (Paganelli et al. 2018).

In a murine gastrointestinal colonization model with systemic β-lactam administration, clade A1 strains dominated over clade B/*E. lactis* strains (Singh et al. 2022). However, in the absence of antibiotics, clade B/*E. lactis* strains outnumbered clade A1 strains and persisted longer in the same model, suggesting that clade A1 strains are replaced by clade B/*E. lactis* strains once the patient leaves the hospital (Montealegre et al. 2016).

While it seems apparent why antibiotic-resistant A1 strains would replace antibiotic susceptible commensal strains, it is an open question whether clade A1 strains can also suppress their growth, or vice versa.

A mode by which bacteria can inhibit the growth of competitors is the release of bacteriocins, and enterococci have been described as one of the most frequent bacteriocin producers (Almeida-Santos et al. 2021). Bacteriocins are defined as ribosomally synthesized antimicrobial proteins or peptides, which either remain unaltered (class II bacteriocins) or are post-translationally modified by biosynthetic enzymes (class I bacteriocins). The biosynthetic gene clusters consist of genes encoding the bacteriocin itself and/or its biosynthetic enzymes, export proteins, producer immunity mechanisms, and, sometimes, regulators of the bacteriocin production (Heilbronner et al. 2021). The antibacterial activities of bacteriocins are very diverse and range from the degradation of essential cellular components to the inhibition of specific molecular targets, such as enzymes required for the synthesis of the cell wall or cell membrane, proteins or nucleic acids, to the disintegration of bacterial membranes, but much of the mode of action of bacteriocins is not fully understood due to their complexity (Simons et al. 2020). Bacteriocin production plays a crucial role in shaping the microbiome, and they can prevent or promote the growth of a bacterial community by another strain or can lead to the redistribution of microbiome members into sub-niches and protect against colonization by bacteriocin-susceptible invaders. This can be utilized in a medical context by using probiotics for pathogen exclusion. In contrast to the majority of current antibiotics, most bacteriocins exhibit a narrow spectrum activity and could thus be attractive agents for precision therapy (Heilbronner et al. 2021).

*Enterococcus*-produced bacteriocins, known as enterocins (Brandis and Brandis 1963), predominantly fall into class II, characterized by their small size (<10 kDa) and heat stability. Within class II, further subdivisions include class IIa (e.g. enterocins A and P), class IIb (e.g. enterocin C, 1071, and X), class IIc (e.g. bacteriocin AS-48 and enterocin 4), and class IId (e.g. Enterocin Q and L50) (Almeida-Santos et al. 2021). Enterocin producers have been studied in different contexts, especially against food-borne pathogens, and have been proposed as probiotics, however, safety concerns have been raised (Hanchi et al. 2018). It has also been discussed that bacteriocins may offer therapeutic options, either alone or in combination with other antimicrobials (Almeida-Santos et al. 2021). Case studies illustrate the feasibility of using bacteriocins against vancomycin-resistant enterococci (VRE) (Phumisantiphong et al. 2017, Bucheli et al. 2022). The diversity, classification, potential use, and concerns regarding enterocins have been described in detail in a recent review article (Almeida-Santos et al. 2021).

Previous studies on interactions between *E. faecium* clades used a limited number of isolates to represent the different clades, thus it is unknown how generalizable the observations are. Here, we used a large collection of 125 isolates, representing the clinical clade A1 and the commensal clade A2 and *E. lactis*, to study the interactions between clinical and commensal isolates.

## Materials and methods

### Bacterial strain collection

**Figure 1.**
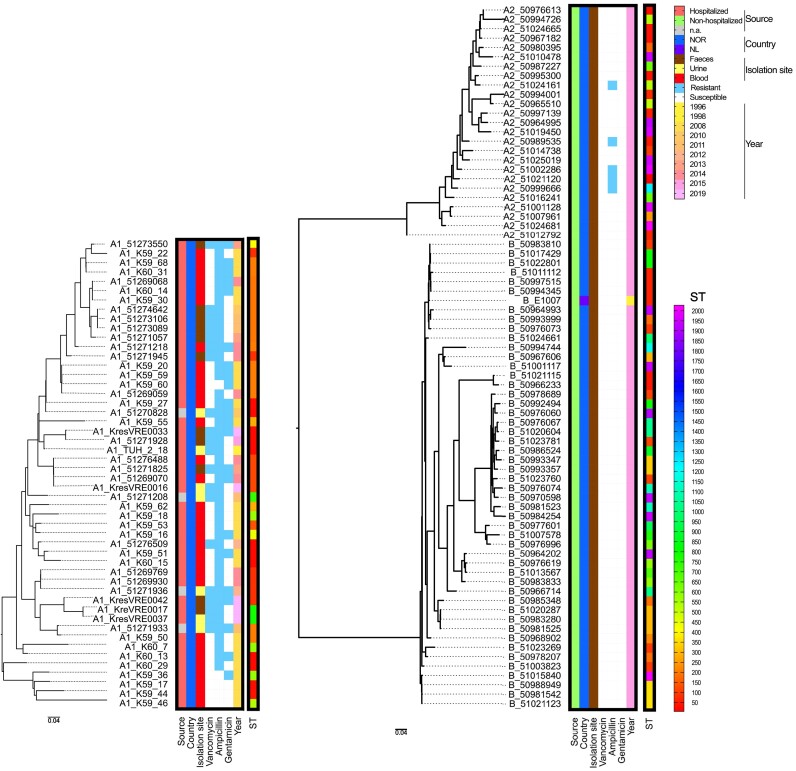
Phylogenetic relation and characteristics of strains used in this study. The strains are sorted according to their phylogenetic relationships as indicated by the phylogenetic tree on the left. Clades A1, A2, B (*E. lactis*) are indicated in front of the strain name. Characteristics (source, country of origin (NOR: Norway, NL: The Netherland), isolation site, resistance to vancomycin, ampicillin, and gentamicin (blue: presence, white: absence), and year of isolation) are color-coded. STs are also color-coded as indicated on the rainbow scale to the right.

The bacterial strain collection was set up to represent 50 clade A1, 25 clade A2 and 50 *E. lactis* (former clade B) isolates. *E. lactis* and A2 isolates were selected from the Tromsø 7 human faecal adult population sample collection (Norwegian National Advisory Unit on Detection of Antimicrobial Resistance (K-res)) and 1 strain from the Netherlands which has previously been shown to be susceptible to human serum (Paganelli et al. 2018). Clade A1 isolates representing CC17 were selected from the Norwegian Surveillance System for Antibiotic Resistance in Microbes (NORM) 2008 and 2014 collections and Norwegian Vancomycin-resistant *E. faecium* (VRE*fm*) collection from 2010 to 2015 (AL-Rubaye et al. 2023) as well as VRE isolates from 2019 received at K-res. The isolates were chosen to represent a wide range of sequence types (ST) and to cover the species phylogeny. Detailed information on the bacterial strain collection is given in Fig. 1 and Supplementary Table S1.

### Whole genome sequencing and bioinformatics analyses

All isolates were genome sequenced and are available under PRJNA858233, PRJEB64173, PRJEB71064 and PRJEB71065 (Table S1). While most clade A1 isolates were Illumina sequenced, the clade A2 and B genome data were mostly hybrid assemblies of Illumina and PacBio sequences. For comparative phylogenetic analyses, separate phylogenies were built for single clade A1 (I), clades A2 and B (II), and all clades combined (III), using K59-59 (PRJNA858233) as a reference for I and III and T7EF-50994744 (PRJEB71065) for II. Mlplasmids v.2.1.0 (Arredondo-Alonso et al. 2018) was used to identify chromosomal contigs in the hybrid assembly of each reference. Sequence reads were then mapped to the reference chromosome using Snippy v.4.6.0 (https://github.com/tseemann/snippy) and SNPs identified using snp-sites v.2.5.1 (Page et al. 2016). Within the core genome with 2 883,282 bp (I and III) and 2 662,653 bp (II) of reference lengths, 13923 (I), 136938 (II) and 120697 (III) SNPs were identified. Maximum-likelihood phylogenies were inferred using RAxML v.8.2.12 with GTR + Gamma rate heterogeneity model and 100 bootstraps (Stamatakis 2014).

### Spot on lawn assay

#### Spot on lawn screening

For the initial interaction screening, the indicator was applied to a brain heart infusion (BHI) plate as a lawn and the putative inhibitor was added on top. For this, the indicator was picked from a blood agar plate and diluted to 0.5 McFarland in 5 ml 0.85% NaCl then diluted 1:10 in 5 ml 0.85% NaCl. This solution was applied with a cotton swab onto a BHI agar plate using a spiral plater for rotating the plate and dried for 5 min before applying the inhibitor. The bacterial solution in NaCl equals 3 × 10^6^ ± 7 × 10^5^ CFU/ml (determined by CFU count n=9) and since a cotton swab takes about 150 mg of liquid, 5 ± 1 × 10^5^ CFU/plate is applied. The inhibitor was grown overnight in 5 ml liquid BHI at 37°C with 220 rpm shaking, and 10 µl of this culture was applied on top of the lawn. Inhibition was read after 18 ± 2 h of incubation at 37°C, according to the scoring illustrated in Fig. S1: 3 – definite inhibition with a wide zone (Inhibition ++), 2 – definite inhibition with an intermediate zone (Inhibition +), 1 – definite inhibition with a narrow zone (Inhibition), 0.5 – non-definite inhibition zone with colonies growing within the zone (Undefined inhibition), 0 – no inhibition.

The average inhibition score per strain was calculated as the sum of inhibition (0 to 3) divided by the number of interactions.

#### Supernatant on lawn

The supernatant of strains that scored 1 in at least 2 interactions was used for further assays. Three representative strains of each clade were used as indicators and plated out as a lawn. Supernatants were obtained by centrifuging 20 ml overnight cultures at 7000 rpm for 10 min at 4°C, supernatants were filtrated through a 0.45 µm syringe filter followed by a 0.2 µm syringe filter (PES, VWR, US) to avoid clogging of the 0.2 µm filter. Sterile supernatants were concentrated 5 times their volume using a 3 MWCO filter (Vivaspin 20, Merck, Germany) at 7000 rpm. Sterile filtrated concentrated supernatants were treated with proteinase K (ThermoFisher Scientific, US) (3 µl of 10 mg/ml in 15 µl supernatant for 2 h at 37°C) or heat-inactivated proteinase K (100°C for 10 min) or heat (100°C for 10 min). 10 µl of untreated and treated supernatant was applied on top of the lawn and inhibition was read after 18 ± 2 h of incubation at 37°C, according to the scoring described (Fig. S1). The supernatant of strains that showed at least one grade 3 inhibition despite proteinase K treatment, was investigated further. One ml of the indicator at 0.5 McFarland was added in 10 ml top-agar (0.5% agar, Sigma Aldrich, US, in BHI), which was poured on a BHI agar plate and dried for 30 min. Tenfold dilutions of the supernatants in PBS (10^−1^ to 10^−8^) were added on top and the plates were read after 18 ± 2 h of incubation at 37°C. PHASTER (PHAge Search Tool Enhanced Release) (Arndt et al. 2016) was used to identify and annotate prophage sequences within the genomes of the 11 strains, which showed at least one grade one inhibition after proteinase K treatment.

### Prediction of bacteriocins using a novel database

The whole genome sequences (WGS) of all strains were screened against a bacteriocin database that has recently been published (Tedim et al. 2023) using CGE MyDbFinder with a cut-off of 80% identity and 80% coverage. Briefly, this bacteriocin database includes 76 Bacillota bacteriocins (mostly from enterococci) that have either been previously described or detected in the process of creating this proprietary database.

### Isolation and characterization of a *ptsD* transposon (Tn) mutant

#### Isolation of a *ptsD* Tn mutant

To study the involvement of *ptsD*, we used a *mariner* Tn mutant library in the strain E8202, as described in (de Maat 2022). A transposon mutant with an insertion in the gene encoding *ptsD* (*ptsD* locus NZ_LR135344.1 711045..711866; E1162 locus tag EfmE1162_1918) was isolated from an *E. faecium* E8202 (hospitalized patient isolate, The Netherlands, 2015, E745) (Top et al. 2020) Tn mutant library (*mariner* Tn cassette carrying a gentamicin (GM) resistance gene) as described previously (Zhang et al. 2017), using the *ptsD*-specific primers ptsd_Efm_Tn_Fw 5′-CGGAAGATGTTTTGGCGCTC-3′ and ptsd_Efm_Tn_Rv 5′-TCCCAAGACGACCATTCCAAA-3′ as well as the bidirectional primer, which is complementary to the repeats flanking the *mariner* Tn sequence, ftp_tn_both_ends_MmeI 5′-CGGGGACTTATCATCCAACC-3′. The mutant *E. faecium* E8202 *ptsD*::GM was first confirmed by in-house Sanger sequencing of the ptsd_Efm_Tn_Rv—ptsd_Efm_Tn_Rv PCR product, and then genome sequenced using the Illumina MiSeq paired-end platform. Genomic comparison of the Tn mutant and wild-type was performed by using Nullarbor v.2.0.20191013 pipeline (https://github.com/tseemann/nullarbor), resulting in one synonymous core SNP between the strains (pos 855 132, CGC to CGA, silent (both arginine)). Abricate v.1.0.1 with a custom database and a minimum identity and coverage of 60% was used in confirming the presence of the insertion only in the mutant. The genomes were further compared with pgv-mauve v.0.3.2 to exclude any other genomic differences.

#### Characterization of the *ptsD* Tn mutant

Overnight cultures of the wildtype strain E8202 and its mutant E8202 *ptsD*::GM were diluted 1:100 in BHI, BHI with 2 g/L mannose, Müller Hinton (MH) broth, MH with 2 g/L mannose (Sigma-Aldrich, US), Lysogeny broth (LB) or LB with 2 g/L mannose and growth was measured in an Epoch 2 Spectrophotometer with Gen5 Software (BioTek Instruments Inc., Winooski, Vermont US) at 37°C, shaking at 425 rpm, with OD_600_ measurement every 10th min for 18 h (n=6, biological triplicates, technical duplicates). E8202 and its mutant E8202 *ptsD*::GM were subjected to the spot on lawn assay as described above and in addition to BHI, the assay was also conducted on BHI with 2 g/L mannose, LB, and MH agar plates.

## Results

### Selection of representative strains

The strains were chosen to represent the diversity of clades A1, A2 (*E. faecium*), and B (*E. lactis*), as shown in Fig. 1. All A1 strains originate from clinical samples, while all A2 and B strains are from faeces of non-hospitalized individuals. Detailed strain characteristics are given in Table S1. A1 is the least diverse clade, while *E. lactis* shows the highest branching.

### Clinical A1 strains inhibit commensal strains

**Figure 2.**
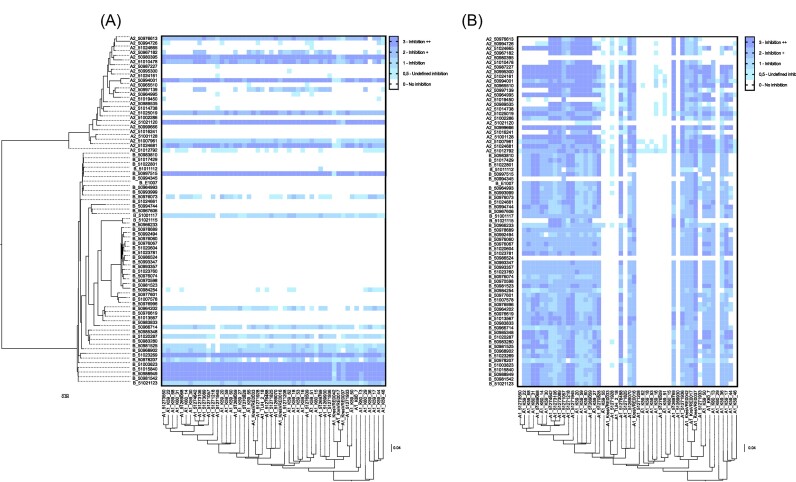
Inhibitory properties of the clades. (A) Clade A1 used as target lawn (x-axis) for investigating inhibition by clade A2 and B (*E. lactis*) strains (y-axis). (B) Clade A2 and B (*E. lactis*) strains used as target lawn (y-axis) for investigating inhibition by A1 strains (x-axis). Interaction is rated as indicated on the right.

**Figure 3.**
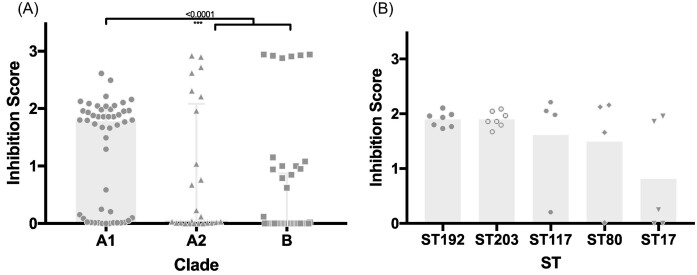
Comparison of inhibition scores. (A) Inhibition score comparison between clade A1, A2, and B (*E. lactis*). (B) Inhibition scores of the clinically relevant STs of clade A1 with n>3. Bars show medians with interquartile range. Statistical analysis was performed using the Kruskal–Wallis test.

Spot on lawn assays were conducted with overnight cultures of one clade and lawns of another clade. These spot on lawn assays showed that clade A1 strains inhibited the growth of clade A2 and B (*E. lactis*) strains to a higher degree than vice versa (Fig. 2, 3). While only 36% of commensal isolates (48% of A2 and 30% of B/*E. lactis*) could inhibit A1 strains, 76% of the clinical A1 strains could inhibit commensal strains. The sum of inhibition values (giving each interaction a value from 0 to 3) for A1 strains was 4742, while it was 2333 for commensal strains. The strains which showed the highest inhibition score belonged to ST5, ST165, ST69, ST2027, and ST1940 in A2, to ST2016, ST60, ST361 and ST96 for *E. lactis* and to ST787, ST78, ST117, ST80, ST192, and ST203 for A1. Phylogenetically related strains show a similar inhibition pattern. The highest inhibition score of clade A1 strains was mediated by the *E. lactis* (clade B) 51003823 ST94 and 51015840 ST2016 as well as clade A2 strains 50980395 ST165 and 51021120 ST5. The highest inhibition score of commensal strains was mediated by the clade A1 strains 51269070 ST117 and 51273089 ST192 (Fig. 2, Table S1).

Average inhibition scores were calculated as the average inhibition per interaction. Clade A1 inhibited commensal strains at a significantly higher level (Fig. 3). Strains that showed at least one grade 2 inhibition were used for further investigation, that is 35 out of 50 A1 strains, 10 out of 25 A2 strains, 16 out of 50 B/*E. lactis*.

### Inhibition is mediated by secreted, heat-stable, proteinaceous compounds

**Figure 4.**
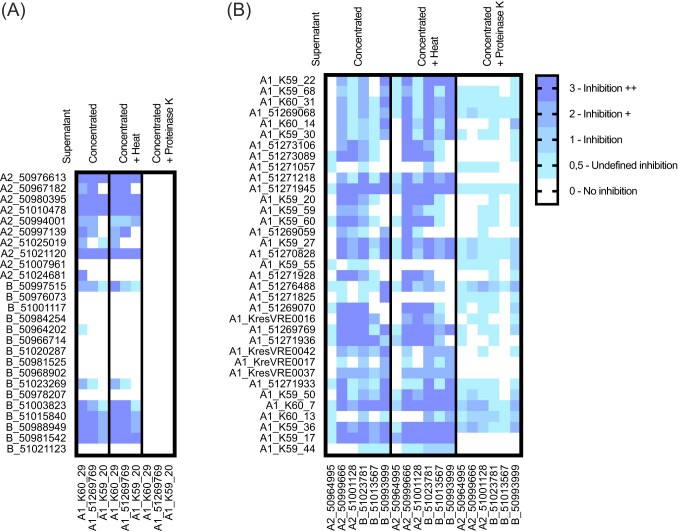
Inhibition mediated by supernatant. (A) Clade A1 strains were used as target lawns (x-axis) and supernatants of clade A2 and B (*E. lactis*) strains (y-axis) were placed on top to investigate their ability to inhibit the target. (B) Clade A2 and B (*E. lactis*) strains (x-axis) were used as target lawns and supernatants of clade A1 strains (y-axis) were placed on top. Supernatants were 5x concentrated, or 5x concentrated and exposed to heat or 5x concentrated and treated with proteinase K. Inhibition is rated as indicated.

Whether supernatants were responsible for the inhibition seen in the initial spot on lawn assay screening was evaluated. Five times concentrated supernatants of all strains, which showed at least one grade 2 inhibition in the assay using overnight culture were used on lawns of three strains representing A1, A2, and B (Fig. 4).

The concentrated supernatants exhibited a similar inhibitory activity as the bacterial overnight culture. Most inhibition was observed mediated by the supernatant of clade A1 strains compared to the other clades. As a control, the flow through from concentration (proteins < 3 kDa) was also tested on the lawn, yielding no observable inhibition. Among the concentrated supernatants, strains 51271945 ST78, K59-17 ST22, and K60-7 ST578 from clade A1 exhibited the highest inhibition scores. Notably, the concentrated supernatants were mostly heat stabile but susceptible to proteinase K treatment (2 mg/ml for 2 h at 37°C). It is noteworthy that a gentler proteinase K treatment (1 mg/ml for 1 h at 37°C, see Fig. S2) did not eliminate the inhibitory effect. Upon heat-inactivation of proteinase K (10 min at 100°C), the inhibitory activity was restored (see Fig. S2).

The inhibition mediated by concentrated supernatants of clade A1 strains against clade A2 and B strains could not be fully abolished by proteinase K treatment, indicating the potential involvement of non-proteinaceous compounds. The strains 51276488 ST117, K59-36 ST575, K60-7 ST578 and K60-14 ST192 showed grade two inhibition even after proteinase K treatment (2 mg/ml for 2 h at 37°C). Hence, we suspect the involvement of phages or other agents that are not sensitive to serine proteases. In silico analysis in Phaster showed that these strains carry one intact prophage region each (Fig. S3). Out of the other nine strains which showed at least one grade one inhibition after proteinase K treatment, three strains did not contain prophage regions (51269068, K59-55 and K60-14), four contained one prophage region each (51269070: Entero_phiFL1A, 51269769: Lister_2389 (Pope et al. 2007), K59-50: Lister_2389, K60-13: Entero_IME_EFm5 (Gong et al. 2016)) and two contained multiple prophage regions (K59-27: Entero_IME_EFm5 (Gong et al. 2016); Entero_phiFL1A (Yasmin et al. 2010), 51270828: Bacill_phBC6A52 (Bruce et al. 2021); Entero_IME_EFm5; Staphy_SPbeta_like (Oliveira et al. 2019)). Since we suspected that phages could be involved in the phenotype of the supernatants of 51276488, K59-36 and K60-7, we expected that single plaques would be visible upon dilution of the native concentrated supernatants. However, 10^−1^ dilution showed grade 1 inhibition, while dilutions 10^−2^ to 10^−8^ showed no inhibition (data not shown). Still, we cannot exclude the involvement of phages and further investigation would be needed.

### Bacteriocin presence in the different clades

**Figure 5.**
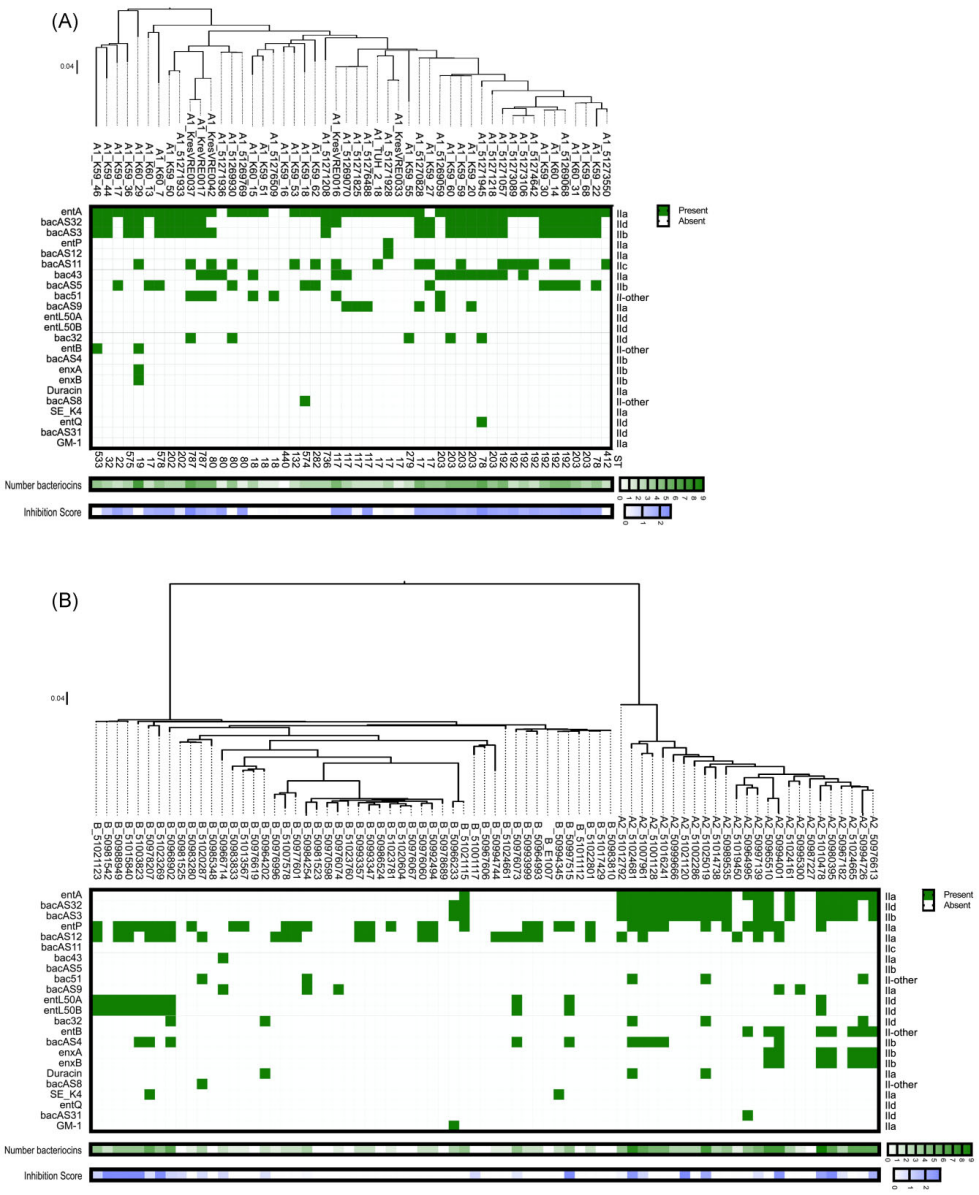
Presence of bacteriocins in genomes of clade A1, A2 and B (*E. lactis*) strains. (A) Bacteriocins in clade A1, (B) Bacteriocins in clade A2 and B (*E. lactis*). Bacteriocin presence was predicted using a novel database. Bacteriocin classes are indicated on the right side of the panel. Total number of bacteriocins is given below, where dark green is the highest number of different bacteriocins (n = 9). The inhibition score from the initial spot on lawn assay is shown at the bottom of the graph, where dark blue is the highest average inhibition score. STs are indicated for clade A1 strains below the panel.

A novel bacteriocin database (Tedim et al. 2023) was used to predict bacteriocin encoding genes in all genomes (Fig. 5). A total of 21 different bacteriocins was detected, including the previously described bacteriocin encoding genes *entA* (Aymerich et al. 1996, Fugaban et al. 2021), *entP* (Cintas et al. 1997), *bac43* (Todokoro et al. 2006), *bac51* (Yamashita et al. 2011), *entL50AB* (Cintas et al. 1998), *bac32* (Inoue et al. 2006), *entB* (Casaus et al. 1997), *enxAB* (Hu et al. 2010), *duracin* (Peeva et al. 2006, Cui et al. 2012), *entSE-K4* (Eguchi et al. 2001), *entQ* (Cintas et al. 2000, Criado et al. 2006a,b), *GM-1* (Kang and Lee 2005) (in order of prevalence across all 125 strains), and nine putative novel bacteriocins (Tedim et al. 2023). Clades A1 and A2 encoded significantly more bacteriocin genes than clade B (*E. lactis*) (Fig. S4). There was a positive correlation between the number of bacteriocins and the inhibition score (Nonparametric Spearman correlation r = 0.563; confidence interval 95%: 0.426 to 0.675, *P* value two-tailed <0.0001, number of XY pairs: 125).

Strains that showed high inhibition and contained many bacteriocin genes could be good candidates for antibiotic development, specifically against A1 or as probiotic strains. Commensal strains carrying *entB, enxA* and *enxB* or *entL50A* and *entL50B* all inhibited clade A1 strains, and *entL50AB, bacAS4* and *duracin* are exclusive to commensal strains in this strain collection. The commensal A2 strain 51010478 (*entA, bacAS32, bacAS3, entP, entL50AB, entB, enxAB*) stands out as a good probiotic candidate since it showed high inhibition to clinical strains and the combination of *entL50AB* with *entB* and *enxAB* is unique to this strain. The A2 strain 50976613 also showed high inhibition and high bacteriocin count, however, its bacteriocin gene profile is the same as 51024665, which does not show any inhibition, thus the predicted bacteriocins of 50976613 are most likely not responsible alone for the observed inhibition phenotype.

Clinical strains that show high inhibition and contain many bacteriocin genes can indicate which bacteriocins are clinically relevant. The bacteriocin genes *bacAS11, bacAS5, entQ*, and *bac43* are exclusive to clade A1 and correlate with an inhibition phenotype (clade B/*E. lactis* strain 50966714 has a 98.67% identical variant of *bac43* but does not show inhibition in supernatant assay). The gene encoding bacteriocin 43 is often co-present with the plasmid replicase *rep_18b_* (10 *_bac43_*_and *rep18b*_/14 *_bac43_*, strains with *bac43* but without *rep_18b_* are 51273106, 51269059, K60-15 and KresVRE0016). Clades A1 and A2 share *entB* and *enxAB*, while A2 and B/*E. lactis* share *bacAS4* and *duracin*, but none of the bacteriocin genes found in A1 and B/*E. lactis* were absent in A2. The genes *bacAS3, bac32* and *bac51* also correlated with an inhibition phenotype and were found in clinical and commensal strains. *BacAS31* was unique to A2, while *bacAS8, entSE-K4* and *GM-1* were unique to clade B/*E. lactis*.

A number of strains showed inhibition in the spot on lawn assay, but only few bacteriocin genes were detected, such as clade A1 strains 51269769 (*entA*), 51271936 (*entA*), 51274642 (*entA* and *bacAS11*), 51273089 (*entA* and *bacAS11*), 51276488 (*entA* and *bacAS9*) and K60-13 (*entA, bacAS5*) and clade B/*E. lactis* strains 50966714 (*bac43* (with 98.67% identity), *bac51*) and 50981542 (*entL50A* and *B*). In these strains, undiscovered bacteriocins and/or other factors could be responsible for the inhibition phenotype.

The two strains, clade B/*E. lactis* strains 51001117 and 50981525, showed inhibition in the spot on lawn interaction screening, but no bacteriocin genes were found. Of note, neither of the strains showed inhibition in the supernatant assay. Thus, certain cues might be necessary for their inhibition phenotype or other factors might be involved.

### The potential role of *ptsD* in susceptibility to inhibitors

Given that some mannose-specific phosphotransferase systems (PTS) have been documented as a receptors for class II bacteriocins and bacteriophages in various bacterial species (Jeckelmann and Erni 2020), and considering that a mannose PTS encoded by *ptsD* and exclusive to clinical strains (referred to as *pts*^Clin^) of *E. faecium* has been identified (Zhang et al. 2013), we proceeded to explore the involvement of *ptsD* in the observed inhibition. All A1 strains, except K59-44 and K59-46, carried *ptsD*, while none of the Clade A2 or B strains carried this gene (Fig. S5). Therefore the Tn mutant E8202 *ptsD*::GM was isolated from an E8202 Tn mutant library and confirmed by WGS. E8202 and its mutant E8202 *ptsD*::GM did not show significant growth differences in BHI, BHI with 2 g/L mannose, LB, LB with 2 g/L mannose, MH or MH with 2 g/L mannose (Fig. S6). They also did not show a difference in susceptibility to inhibition by other A1, A2 or B strains in spot on lawn assays (Fig. S7).

## Discussion

Our results show that clade A1 isolates can suppress the growth of commensal isolates. This may help to understand the dynamics of clade A1 carriage preceding infection in a nosocomial setting. Our findings are in line with the results from a murine gastrointestinal colonization model with systemic β-lactam administration where clade A1 strains dominated over *E. lactis* (clade B) strains (Singh et al. 2022) and in the absence of antibiotics, *E. lactis* (clade B) outnumbered A1 strains (Montealegre et al. 2016). Of note, the strength of these two studies is the use of a murine gastrointestinal colonization model, however, they only used a total of 12 strains and the use of antibiotics may limit the comparability to our findings. While many previous studies were biased towards either clinical multidrug-resistant (MDR) *E. faecium* strains or non-human environmental *E. faecium* strains or were limited in strain number, the strength of our study is the balance of human clinical isolates and human commensal isolates and the high number of isolates.

In the spot on lawn screening the cell density of the spot is much higher than the lawn, which represents the conditions of overgrowth. Here, we found that nosocomial clade A1 strains inhibit commensal strains significantly more than the other way around. With only 3 exceptions, all other 72 commensal strains are susceptible to inhibition by multiple nosocomial clade A1 strains, which can partially explain the predominance of these in infections. Three of the nosocomial ST192 strains which show high inhibition belong to the core genome MLST cluster type 3 which has been described as a successful clone in Norway (AL-Rubaye et al. 2023). The A1 STs showing inhibition of commensal strains are known as invasive in Europe (Werner et al. 2020, AL-Rubaye et al. 2023). Interestingly, we also found several commensal strains, which can inhibit all clinical strains, including VRE and other MDR strains. These strains have high potential for use as probiotics. Moreover, since we also show that many commensal strains lack the ability to inhibit nosocomial strains, probiotic strains should be chosen carefully after phenotypic characterization to fully harness their potential. It is a limitation of this study that cell-to-cell contact or other cues (Gonzalez and Mavridou 2019) which might be required to trigger the phenotype of some interactions was not considered. Pilot experiments where both strains were grown together, and the supernatant from this co-culture was isolated, did not show inhibition. The presumable reason is that the supernatant represents a mixture of secretions of both strains and thus also putative immunity proteins, as well as a dilution of putative effectors. Future studies could investigate whether certain stressors can enhance interactions.

Since the interactions were mostly mediated by heat-stable proteinaceous secreted compounds, the presence of bacteriocins in the genome sequences was predicted using a novel database (Tedim et al. 2023). We observed that clades A1 and A2 encoded significantly higher numbers of bacteriocins compared to *E. lactis*. The number of bacteriocins is in line with the results from our interaction screening, where clinical strains inhibit commensal strains significantly more often than the other way around. Many of the strains showing a high inhibition phenotype also encoded a high number of bacteriocins, such as *entA* (Aymerich et al. 1996, Fugaban et al. 2021), *bac43* (Todokoro et al. 2006), *entP* (Cintas et al. 1997), *bac32* (Inoue et al. 2006), *bac51* (Yamashita et al. 2011) and *entL50A* and *B* (Cintas et al. 1998). All known predicted bacteriocins were of Class II, meaning ribosomally synthesized antimicrobial proteins or peptides, which do not undergo posttranslational modification. In addition, nine genes encoding novel bacteriocins, called *bacAS#*, were detected in the strain sequences. The bacteriocin encoding genes *entB, enxA* and *B, entL50A* and *B* were all found in strains able to inhibit clade A1 strains, and *entL50A* and *B* are exclusive to commensal strains. These bacteriocins would thus be good candidates for antimicrobial development or as adjuvants of antibiotics. The bacteriocin encoding genes *bac43, bacAS11* and *bacAS5* were exclusive to clade A1 and co-occurred with an inhibition phenotype. They thus stand out as clinically relevant bacteriocins. Bacteriocin 43 has originally been discovered in a clinical VR*E. faecium* strain from the US and was described as a Class IIa bacteriocin, active against several enterococcal species and located on mobilizable plasmids (Todokoro et al. 2006). It was later also discovered on small theta-replicating plasmids (*rep_18b_*) of different VR*E. faecium* strains from hospitalized patients from Germany and Canada (Freitas et al. 2016). In our strain collection, the *bac43* gene was found in VR*E. faecium* (*vanA* or *vanB*) and in VSE strains and *bac43* was mostly co-present with *rep_18b_* (10 *_bac43_*_and *rep18b*_/14 *_bac43_*) which is in line with the previous findings. Of note, some bacteriocins, such as Enterocin Q (Cintas et al. 2000, Criado et al. 2006a ,b), are optimally produced at higher temperatures and might thus not have been responsible for the phenotype we see in the interaction screening.

It is a limitation of this study, that the regulation and expression of the individual enterocins were not studied. Production, release, and activity of bacteriocins, are tightly controlled by a complex network of genetic elements. For example, the expression of cytolysin is tightly controlled by a two-component regulatory or a quorum-sensing system (Haas et al. 2002). Also, some Class IIa bacteriocins with a double glycine leader, which also includes a few enterocins, are regulated by a three-component regulatory system that encompasses a peptide pheromone, a membrane-bound histidine protein kinase that serves as receptor for the peptide pheromone, and finally, a response regulator protein that activates the operons participating in the bacteriocin biosynthesis upon phosphorylation (Nes et al. 2014). Future studies could also study the bacteriocin-operons’ immunity genes.

The presence or absence of bacteriocin genes could not explain all phenotypes that we observed in the spot on lawn interaction screening. For example, some strains share the same bacteriocin genes, but show a different inhibition pattern, while others share an inhibition pattern but have differences in their bacteriocin genes. We thus suspect that there might be other bacteriocins or yet other factors than bacteriocins involved. Since some of the inhibition mediated by clade A1 supernatants was not abolished by proteinase K treatment, we suspect the involvement of non-proteinaceous agents, such as lipopeptides or phages, and in silico analysis in Phaster showed that these strains carry an intact prophage region.

A receptor for bacteriocins and bacteriophages across bacterial species is the mannose-specific phosphotransferase system (PTS) which also acts as a mannose transporter (Jeckelmann and Erni 2020). In *E. faecalis*, it has been shown that the *mpt* operon, encoding a mannose phosphotransferase system, is involved in bacteriocin susceptibility (Héchard et al. 2001, Opsata et al. 2010). Bacteriocin resistance of *E. faecalis* was linked to reduced expression of the *mpt* operon, and an *mptD* mutant was bacteriocin (pediocin PA-1 and mesentericin Y105) resistant (Héchard et al. 2001, Opsata et al. 2010). Moreover, mannose-induced PTS expression leads to enhanced sensitivity of *E. faecalis* JH2-2 to the bacteriocin mesentericin Y105 (Héchard et al. 2001). In *E. faecium*, a mannose PTS, encoded by *ptsD* exclusive to clinical strains, is involved in colonization by clinical strains during antibiotic treatment (Zhang et al. 2013, AL-Rubaye et al. 2023) and it was suggested that the mannose PTS system of *E. faecium* could be a mean to control *E. faecium* (Somarajan and Murray 2013). It was also speculated that *ptsD* could be targeted by bacteriocins (Zhang et al. 2013), since mannose PTSs are common targets of bacteriocins (Kjos et al. 2010, Opsata et al. 2010). In our study, we did not observe a difference in susceptibility to inhibition between E8202 and its mutant E8202 *ptsD*::GM. This might be because the secretions of the inhibitors contain multiple compounds and even if one lacks its target the other compounds could still exert the phenotype. In addition, E8202 could compensate for the lack of *ptsD* by the use of another mannose PTS. Potentially, the effect of a compound on *ptsD* can only be seen for isolated compounds at higher concentrations and the spot on lawn assay might have lacked the sensitivity to detect this. Furthermore, it could be that the whole *pts* operon or a different component than *ptsD* is targeted since an extracellular loop of the membrane-located protein MptC was responsible for specific target recognition by the class IIa bacteriocins in *Listeria monocytogenes* (Kjos et al. 2010).

In summary, our study offers critical insights into the dynamics of *E. faecium* colonization within both hospital and community environments. Specifically, in a hospital setting, we predict the displacement of commensal strains by nosocomial clade A1 strains. This replacement is driven by the pronounced ability of clade A1 strains to inhibit clade A2 and B strains and will be propelled in the presence of selective pressure by antimicrobials, where the AMR profile will give clade A1 strains an additional advantage over susceptible clade A2 and B strains. In contrast, in a community setting, we anticipate the replacement of clinical clade A1 strains by commensal strains, as these are also able to inhibit the growth of clade A1 strains. This is likely to be spurred by the carriage of AMR genes in A1, which will pose a fitness cost to the carrier in the absence of antibiotic pressure.

Overall, this study provides insights into the complex interplay of *E. faecium* clades, which has important implications for clinical and public health strategies. Firstly, the implications include the sensible use of antimicrobials to minimize the selective pressure driving the overgrowth and spread of clade A1 strains as well as the need for antimicrobial stewardship programs. Secondly, the predicted displacement of clinical strains by commensal strains in the community underscores the potential benefits of promoting commensal strains to prevent the overgrowth of clinical strains, which could include interventions promoting commensal colonization, such as probiotics or faecal microbiota transplantation. Future research should continue to explore these interactions and the potential interventions our study has suggested.

## Supplementary Material

xtae009_Supplemental_File
