# Supplementary material for: Interactions between commensal Enterococcus faecium and Enterococcus lactis and clinical isolates of Enterococcus faecium
Source: FEMS Microbes. 2024 Mar 9;5:xtae009. doi: 10.1093/femsmc/xtae009 (PMC11008740; doi:10.1093/femsmc/xtae009)
Supplement: xtae009_Supplemental_File [file xtae009_supplemental_file.docx]

Supplementary Table and figures for

“Interactions between commensal *Enterococcus* *faecium* and *Enterococcus* *lactis* and clinical isolates of *E.* *faecium*”

Theresa Maria Wagner, Anna Kaarina Pöntinen, Carolin Kornelia Fenzel, Daniel Engi, Jessin Janice,

Ana C. Almeida-Santos, Ana P. Tedim, Ana R. Freitas, Luísa Peixe, Willem van Schaik,

Mona Johannessen^a^, Kristin Hegstad

**Table S1.** Strains used in this study and their characteristics.

| Strain name | Species | ST | *van* type | Ampicillin R | Gentamicin R | Year | Source | Genome/genbank | Biosample | Bioproject |
| --- | --- | --- | --- | --- | --- | --- | --- | --- | --- | --- |
| A1_51273550 | *E. faecium* | 412 | *vanA* | n.a. | n.a. | 2013 | Faeces | SAMEA114565947 | ERX11596628 | PRJEB64173 |
| A1_K59_22 | *E. faecium* | 78 | none | Yes | No | 2008 | Blood | JANEQB000000000 | SAMN29681728 | PRJNA858233 |
| A1_K59_68 | *E. faecium* | 203 | none | Yes | Yes | 2008 | Blood | GCA_002263115.1 | SAMN07326775 | PRJNA393251 |
| A1_K60_31 | *E. faecium* | 203 | none | Yes | Yes | 2008 | Blood | JANEMY000000000 | SAMN29681809 | PRJNA858233 |
| A1_51269068 | *E. faecium* | 192 | none | Yes | Yes | 2014 | Blood | JANEVX000000000 | SAMN29681575 | PRJNA858233 |
| A1_K60_14 | *E. faecium* | 192 | none | Yes | Yes | 2008 | Blood | JANENO000000000 | SAMN29681793 | PRJNA858233 |
| A1_K59_30 | *E. faecium* | 192 | none | Yes | No | 2008 | Blood | JANEPU000000000 | SAMN29681735 | PRJNA858233 |
| A1_51274642 | *E. faecium* | 192 | *vanB* | Yes | No | 2010 | Feces | JANERK000000000 | SAMN29681693 | PRJNA858233 |
| A1_51273106 | *E. faecium* | 192 | *vanB* | Yes | No | 2011 | Feces | JANEUN000000000 | SAMN29681612 | PRJNA858233 |
| A1_51273089 | *E. faecium* | 192 | *vanB* | Yes | No | 2011 | Feces | JANEVE000000000 | SAMN29681595 | PRJNA858233 |
| A1_51271057 | *E. faecium* | 192 | *vanA* | Yes | No | 2013 | Feces | JANEVP000000000 | SAMN29681583 | PRJNA858233 |
| A1_51271218 | *E. faecium* | 203 | *vanA* | Yes | Yes | 2014 | Blood | JANEVO000000000 | SAMN29681584 | PRJNA858233 |
| A1_51271945 | *E. faecium* | 78 | *vanA* | Yes | No | 2014 | Urine | JANDNZ000000000 | SAMN29678926 | PRJNA858233 |
| A1_K59_20 | *E. faecium* | 203 | none | Yes | Yes | 2008 | Blood | JANEQD000000000 | SAMN29681726 | PRJNA858233 |
| A1_K59_59 | *E. faecium* | 203 | none | Yes | Yes | 2008 | Blood | JANEOV000000000 | SAMN29681760 | PRJNA858233 |
| A1_K59_60 | *E. faecium* | 203 | none | No | Yes | 2008 | Blood | JANEOU000000000 | SAMN29681761 | PRJNA858233 |
| A1_51269059 | *E. faecium* | 203 | none | Yes | No | 2014 | Blood | JANEVY000000000 | SAMN29681574 | PRJNA858233 |
| A1_K59_27 | *E. faecium* | 17 | none | Yes | Yes | 2008 | Blood | JANEPX000000000 | SAMN29681732 | PRJNA858233 |
| A1_51270828 | *E. faecium* | 17 | *vanB* | Yes | Yes | 2010 | Urine | JANEVQ000000000 | SAMN29681582 | PRJNA858233 |
| A1_K59_55 | *E. faecium* | 279 | none | Yes | Yes | 2008 | Blood | JANEOZ000000000 | SAMN29681756 | PRJNA858233 |
| A1_KresVRE0033 | *E. faecium* | 17 | *vanB* | n.a. | n.a. | 2019 | Faeces | ERX11596625 | SAMEA114565948 | PRJEB64173 |
| A1_51271928 | *E. faecium* | 17 | *vanA* | Yes | No | 2015 | Feces | JANEVJ000000000 | SAMN29681590 | PRJNA858233 |
| A1_TUH_2_18 | *E. faecium* | 17 | *vanB* | Yes | No | 1996 | Urine | JANEMP000000000 | SAMN29681819 | PRJNA858233 |
| A1_51276488 | *E. faecium* | 117 | none | Yes | No | 2014 | Blood | JANERI000000000 | SAMN29681695 | PRJNA858233 |
| A1_51271825 | *E. faecium* | 117 | *vanB* | Yes | Yes | 2013 | Feces | JANEVL000000000 | SAMN29681587 | PRJNA858233 |
| A1_51269070 | *E. faecium* | 117 | none | Yes | Yes | 2014 | Blood | JANDVH000000000 | SAMN29678736 | PRJNA858233 |
| A1_KresVRE0016 | *E. faecium* | 117 | *vanB* | Yes | No | 2019 | Urine | ERX11596623 | SAMEA114565945 | PRJEB64173 |
| A1_51271208 | *E. faecium* | 736 | *vanA* | Yes | No | 2011 | Urine | JANDRF000000000 | SAMN29678842 | PRJNA858233 |
| A1_K59_62 | *E. faecium* | 282 | none | Yes | No | 2008 | Blood | JANEOT000000000 | SAMN29681762 | PRJNA858233 |
| A1_K59_18 | *E. faecium* | 574 | none | Yes | No | 2008 | Blood | JANEQF000000000 | SAMN29681724 | PRJNA858233 |
| A1_K59_53 | *E. faecium* | 132 | none | Yes | No | 2008 | Blood | JANEPB000000000 | SAMN29681754 | PRJNA858233 |
| Strain name | **Species** | **ST** | ***van* type** | **Ampicillin R** | **Gentamicin R** | **Year** | **Source** | **Genome/GenBank** | **Biosample** | **Bioproject** |
| A1_K59_16 | *E. faecium* | 440 | none | Yes | Yes | 2008 | Blood | JANEQH000000000 | SAMN29681722 | PRJNA858233 |
| A1_51276509 | *E. faecium* | 18 | *vanA* | Yes | No | 2012 | Blood | JANEQT000000000 | SAMN29681710 | PRJNA858233 |
| A1_K59_51 | *E. faecium* | 18 | none | Yes | Yes | 2008 | Blood | JANEPD000000000 | SAMN29681752 | PRJNA858233 |
| A1_K60_15 | *E. faecium* | 18 | none | Yes | No | 2008 | Blood | JANENN000000000 | SAMN29681794 | PRJNA858233 |
| A1_51269769 | *E. faecium* | 80 | none | Yes | No | 2014 | Blood | JANEVS000000000 | SAMN29681580 | PRJNA858233 |
| A1_51269930 | *E. faecium* | 80 | none | Yes | No | 2014 | Blood | JANEVR000000000 | SAMN29681581 | PRJNA858233 |
| A1_51271936 | *E. faecium* | 80 | *vanA* | Yes | Yes | 2012 | Urine | JANEVH000000000 | SAMN29681592 | PRJNA858233 |
| A1_KresVRE0042 | *E. faecium* | 80 | *vanA* | Yes | Yes | 2019 | Faeces | ERX11596627 | SAMEA114565950 | PRJEB64173 |
| A1_KresVRE0017 | *E. faecium* | 787 | *vanB* | Yes | No | 2019 | Faeces | ERX11596624 | SAMEA114565946 | PRJEB64173 |
| A1_KresVRE0037 | *E. faecium* | 787 | *vanB* | Yes | No | 2019 | Urine | ERX11596626 | SAMEA114565949 | PRJEB64173 |
| A1_51271933 | *E. faecium* | 202 | *vanA* | Yes | Yes | 2011 | Urine | JANEVI000000000 | SAMN29681591 | PRJNA858233 |
| A1_K59_50 | *E. faecium* | 202 | none | Yes | No | 2008 | Blood | JANEPE000000000 | SAMN29681751 | PRJNA858233 |
| A1_K60_7 | *E. faecium* | 578 | none | Yes | No | 2008 | Blood | JANENU000000000 | SAMN29681787 | PRJNA858233 |
| A1_K60_13 | *E. faecium* | 17 | none | Yes | Yes | 2008 | Blood | JANENP000000000 | SAMN29681792 | PRJNA858233 |
| A1_K60_29 | *E. faecium* | 19 | none | Yes | No | 2008 | Blood | JANENA000000000 | SAMN29681807 | PRJNA858233 |
| A1_K59_36 | *E. faecium* | 575 | none | No | Yes | 2008 | Blood | JANEPO000000000 | SAMN29681741 | PRJNA858233 |
| A1_K59_17 | *E. faecium* | 22 | none | No | No | 2008 | Blood | JANEQG000000000 | SAMN29681723 | PRJNA858233 |
| A1_K59_44 | *E. faecium* | 32 | none | No | No | 2008 | Blood | JANEPI000000000 | SAMN29681747 | PRJNA858233 |
| A1_K59_46 | *E. faecium* | 533 | none | No | No | 2008 | Blood | JANEPH000000000 | SAMN29681748 | PRJNA858233 |
| A2_50976613 | *E. faecium* | 22 | none | No | No | 2015 | Non-hospitalized person |  |  | PRJEB71064 |
| A2_50994726 | *E. faecium* | 533 | none | No | No | 2015 | Non-hospitalized person |  |  | PRJEB71064 |
| A2_51024665 | *E. faecium* | 32 | none | No | No | 2015 | Non-hospitalized person |  |  | PRJEB71064 |
| A2_50967182 | *E. faecium* | 32 | none | No | No | 2015 | Non-hospitalized person |  |  | PRJEB71064 |
| A2_50980395 | *E. faecium* | 165 | none | No | No | 2015 | Non-hospitalized person |  |  | PRJEB71064 |
| A2_51010478 | *E. faecium* | 1940 | none | No | No | 2015 | Non-hospitalized person |  |  | PRJEB71064 |
| A2_50987227 | *E. faecium* | 640 | none | No | No | 2015 | Non-hospitalized person |  |  | PRJEB71064 |
| A2_50995300 | *E. faecium* | 29 | none | No | No | 2015 | Non-hospitalized person |  |  | PRJEB71064 |
| A2_51024161 | *E. faecium* | 524 | none | Yes | No | 2015 | Non-hospitalized person |  |  | PRJEB71064 |
| A2_50994001 | *E. faecium* | 69 | none | No | No | 2015 | Non-hospitalized person |  |  | PRJEB71064 |
| A2_50965510 | *E. faecium* | 512 | none | No | No | 2015 | Non-hospitalized person |  |  | PRJEB71064 |
| A2_50997139 | *E. faecium* | 52 | none | No | No | 2015 | Non-hospitalized person |  |  | PRJEB71064 |
| A2_50964995 | *E. faecium* | 1928 | none | No | No | 2015 | Non-hospitalized person |  |  | PRJEB71064 |
| A2_51019450 | *E. faecium* | 1971 | none | No | No | 2015 | Non-hospitalized person |  |  | PRJEB71064 |
| Strain name | **Species** | **ST** | ***van* type** | **Ampicillin R** | **Gentamicin R** | **Year** | **Source** | **Genome/GenBank** | **Biosample** | **Bioproject** |
| A2_50989535 | *E. faecium* | 44 | none | Yes | No | 2015 | Non-hospitalized person |  |  | PRJEB71064 |
| A2_51014738 | *E. faecium* | 101 | none | No | No | 2015 | Non-hospitalized person |  |  | PRJEB71064 |
| A2_51025019 | *E. faecium* | 1952 | none | No | No | 2015 | Non-hospitalized person |  |  | PRJEB71064 |
| A2_51002286 | *E. faecium* | 1994 | none | Yes | No | 2015 | Non-hospitalized person |  |  | PRJEB71064 |
| A2_51021120 | *E. faecium* | 5 | none | Yes | No | 2015 | Non-hospitalized person |  |  | PRJEB71064 |
| A2_50999666 | *E. faecium* | 1239 | none | Yes | No | 2015 | Non-hospitalized person |  |  | PRJEB71064 |
| A2_51016241 | *E. faecium* | 649 | none | No | No | 2015 | Non-hospitalized person |  |  | PRJEB71064 |
| A2_51001128 | *E. faecium* | 1982 | none | No | No | 2015 | Non-hospitalized person |  |  | PRJEB71064 |
| A2_51007961 | *E. faecium* | 247 | none | No | No | 2015 | Non-hospitalized person |  |  | PRJEB71064 |
| A2_51024681 | *E. faecium* | 2027 | none | No | No | 2015 | Non-hospitalized person |  |  | PRJEB71064 |
| A2_51012792 | *E. faecium* | 59 | none | No | No | 2015 | Non-hospitalized person |  |  | PRJEB71064 |
| B_50983810 | *E. lactis* | 94 | none | No | No | 2015 | Non-hospitalized person |  |  | PRJEB71065 |
| B_51017429 | *E. lactis* | 800 | none | No | No | 2015 | Non-hospitalized person |  |  | PRJEB71065 |
| B_51022801 | *E. lactis* | 800 | none | No | No | 2015 | Non-hospitalized person |  |  | PRJEB71065 |
| B_51011112 | *E. lactis* | 60 | none | No | No | 2015 | Non-hospitalized person |  |  | PRJEB71065 |
| B_50997515 | *E. lactis* | 60 | none | No | No | 2015 | Non-hospitalized person |  |  | PRJEB71065 |
| B_50994345 | *E. lactis* | 60 | none | No | No | 2015 | Non-hospitalized person |  |  | PRJEB71065 |
| B_E1007 | *E. lactis* | 61 | none | No | No | 1998 | Non-hospitalized person | GCA_000321625.1 | SAMN00779868 | PRJNA73043 |
| B_50964993 | *E. lactis* | 1927 | none | No | No | 2015 | Non-hospitalized person |  |  | PRJEB71065 |
| B_50993999 | *E. lactis* | 178 | none | No | No | 2015 | Non-hospitalized person |  |  | PRJEB71065 |
| B_50976073 | *E. lactis* | 94 | none | No | No | 2015 | Non-hospitalized person |  |  | PRJEB71065 |
| B_51024661 | *E. lactis* | 994 | none | No | No | 2015 | Non-hospitalized person |  |  | PRJEB71065 |
| B_50994744 | *E. lactis* | 1191 | none | No | No | 2015 | Non-hospitalized person |  |  | PRJEB71065 |
| B_50967606 | *E. lactis* | 289 | none | No | No | 2015 | Non-hospitalized person |  |  | PRJEB71065 |
| B_51001117 | *E. lactis* | 1938 | none | No | No | 2015 | Non-hospitalized person |  |  | PRJEB71065 |
| B_51021115 | *E. lactis* | 39 | none | No | No | 2015 | Non-hospitalized person |  |  | PRJEB71065 |
| 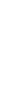B_50966233 | *E. lactis* | 39 | none | No | No | 2015 | Non-hospitalized person |  |  | PRJEB71065 |
| B_50978689 | *E. lactis* | 107 | none | No | No | 2015 | Non-hospitalized person |  |  | PRJEB71065 |
| B_50992494 | *E. lactis* | 798 | none | No | No | 2015 | Non-hospitalized person |  |  | PRJEB71065 |
| B_50976060 | *E. lactis* | 1911 | none | No | No | 2015 | Non-hospitalized person |  |  | PRJEB71065 |
| B_50976067 | *E. lactis* | 1036 | none | No | No | 2015 | Non-hospitalized person |  |  | PRJEB71065 |
| B_51020604 | *E. lactis* | 1036 | none | No | No | 2015 | Non-hospitalized person |  |  | PRJEB71065 |
| B_51023781 | *E. lactis* | 116 | none | No | No | 2015 | Non-hospitalized person |  |  | PRJEB71065 |
| Strain name | **Species** | **ST** | ***van* type** | **Ampicillin R** | **Gentamicin R** | **Year** | **Source** | **Genome/GenBank** | **Biosample** | **Bioproject** |
| B_50986524 | *E. lactis* | 874 | none | No | No | 2015 | Non-hospitalized person |  |  | PRJEB71065 |
| B_50993347 | *E. lactis* | 328 | none | No | No | 2015 | Non-hospitalized person |  |  | PRJEB71065 |
| B_50993357 | *E. lactis* | 328 | none | No | No | 2015 | Non-hospitalized person |  |  | PRJEB71065 |
| B_51023760 | *E. lactis* | 107 | none | No | No | 2015 | Non-hospitalized person |  |  | PRJEB71065 |
| B_50976074 | *E. lactis* | 1101 | none | No | No | 2015 | Non-hospitalized person |  |  | PRJEB71065 |
| B_50970598 | *E. lactis* | 1939 | none | No | No | 2015 | Non-hospitalized person |  |  | PRJEB71065 |
| B_50981523 | *E. lactis* | 1105 | none | No | No | 2015 | Non-hospitalized person |  |  | PRJEB71065 |
| B_50984254 | *E. lactis* | 1945 | none | No | No | 2015 | Non-hospitalized person |  |  | PRJEB71065 |
| B_50977601 | *E. lactis* | 945 | none | No | No | 2015 | Non-hospitalized person |  |  | PRJEB71065 |
| B_51007578 | *E. lactis* | 773 | none | No | No | 2015 | Non-hospitalized person |  |  | PRJEB71065 |
| B_50976996 | *E. lactis* | 623 | none | No | No | 2015 | Non-hospitalized person |  |  | PRJEB71065 |
| B_50964202 | *E. lactis* | 1926 | none | No | No | 2015 | Non-hospitalized person |  |  | PRJEB71065 |
| B_50976619 | *E. lactis* | 583 | none | No | No | 2015 | Non-hospitalized person |  |  | PRJEB71065 |
| B_51013567 | *E. lactis* | 696 | none | No | No | 2015 | Non-hospitalized person |  |  | PRJEB71065 |
| B_50983833 | *E. lactis* | 583 | none | No | No | 2015 | Non-hospitalized person |  |  | PRJEB71065 |
| B_50966714 | *E. lactis* | 1031 | none | No | No | 2015 | Non-hospitalized person |  |  | PRJEB71065 |
| B_50985348 | *E. lactis* | 178 | none | No | No | 2015 | Non-hospitalized person |  |  | PRJEB71065 |
| B_51020287 | *E. lactis* | 296 | none | No | No | 2015 | Non-hospitalized person |  |  | PRJEB71065 |
| B_50983280 | *E. lactis* | 296 | none | No | No | 2015 | Non-hospitalized person |  |  | PRJEB71065 |
| B_50981525 | *E. lactis* | 296 | none | No | No | 2015 | Non-hospitalized person |  |  | PRJEB71065 |
| B_50968902 | *E. lactis* | 218 | none | No | No | 2015 | Non-hospitalized person |  |  | PRJEB71065 |
| B_51023269 | *E. lactis* | 96 | none | No | No | 2015 | Non-hospitalized person |  |  | PRJEB71065 |
| B_50978207 | *E. lactis* | 178 | none | No | No | 2015 | Non-hospitalized person |  |  | PRJEB71065 |
| B_51003823 | *E. lactis* | 94 | none | No | No | 2015 | Non-hospitalized person |  |  | PRJEB71065 |
| B_51015840 | *E. lactis* | 2016 | none | No | No | 2015 | Non-hospitalized person |  |  | PRJEB71065 |
| B_50988949 | *E. lactis* | 361 | none | No | No | 2015 | Non-hospitalized person |  |  | PRJEB71065 |
| B_50981542 | *E. lactis* | 361 | none | No | No | 2015 | Non-hospitalized person |  |  | PRJEB71065 |
| B_51021123 | *E. lactis* | 361 | none | No | No | 2015 | Non-hospitalized person |  |  | PRJEB71065 |

**van* type = vancomycin resistance type; R = resistance


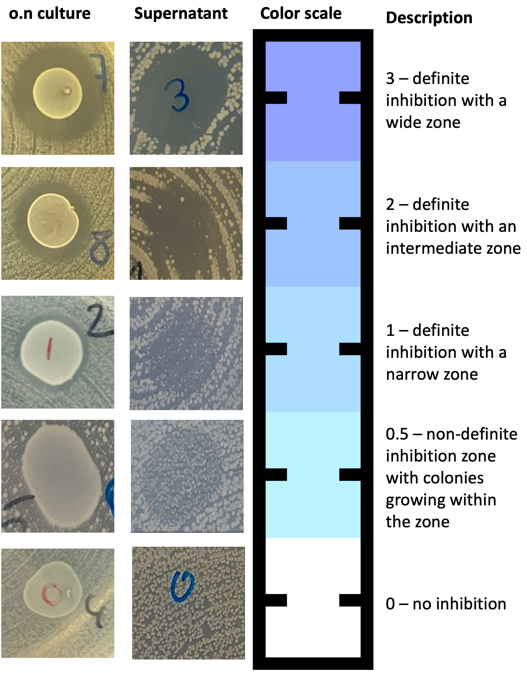


**Figure S1. The scale used to score inhibition.** Inhibition is scored for overnight cultures and supernatant in spot on lawn assays as illustrated.

**Figure S2. Inhibition mediated by 5x concentrated supernatant exposed to heat-inactivated proteinase K.** a and b) Heat-inactivated proteinase K, c and d) proteinase K treatment at 1 mg/ml for 1 h. a and c) Clade A1 strains (x-axis) were used as target lawns and supernatants of clade A2 and B (*E. lactis*) strains (y-axis) were placed on top to investigate their ability to inhibit the target. b and d) Clade A2 and B (*E. lactis*) strains (x-axis) were used as target lawns and supernatants of clade A1 strains (y-axis) were placed on top. Inhibition is rated as indicated.

**Figure S3. Prophage regions as predicted in Phaster.** a) Entero_phiFL1A in strain 51276488 (*Siphoviridae*, with a region length of 32 kb, a score of 120 and 44 total proteins and a GC percentage of 34.56%) , b) Entero_phiFL1A in strain K60-7 (*Siphoviridae*, with a region length of 25.1 kb, a score of 110 and 41 total proteins and a GC percentage of 35.5%), c) Lister_2389 in strain K59-36 (*Caudoviricetes*, with a region length of 30.7 kb, a score of 100 and 49 total proteins and a GC percentage of 35.87%).

**Figure S4. Comparison of bacteriocin count between clade A1, A2 and B (*E. lactis*).** Bars show medians with interquartile range. Statistical P-values were calculated with Kruskal-Wallis test.


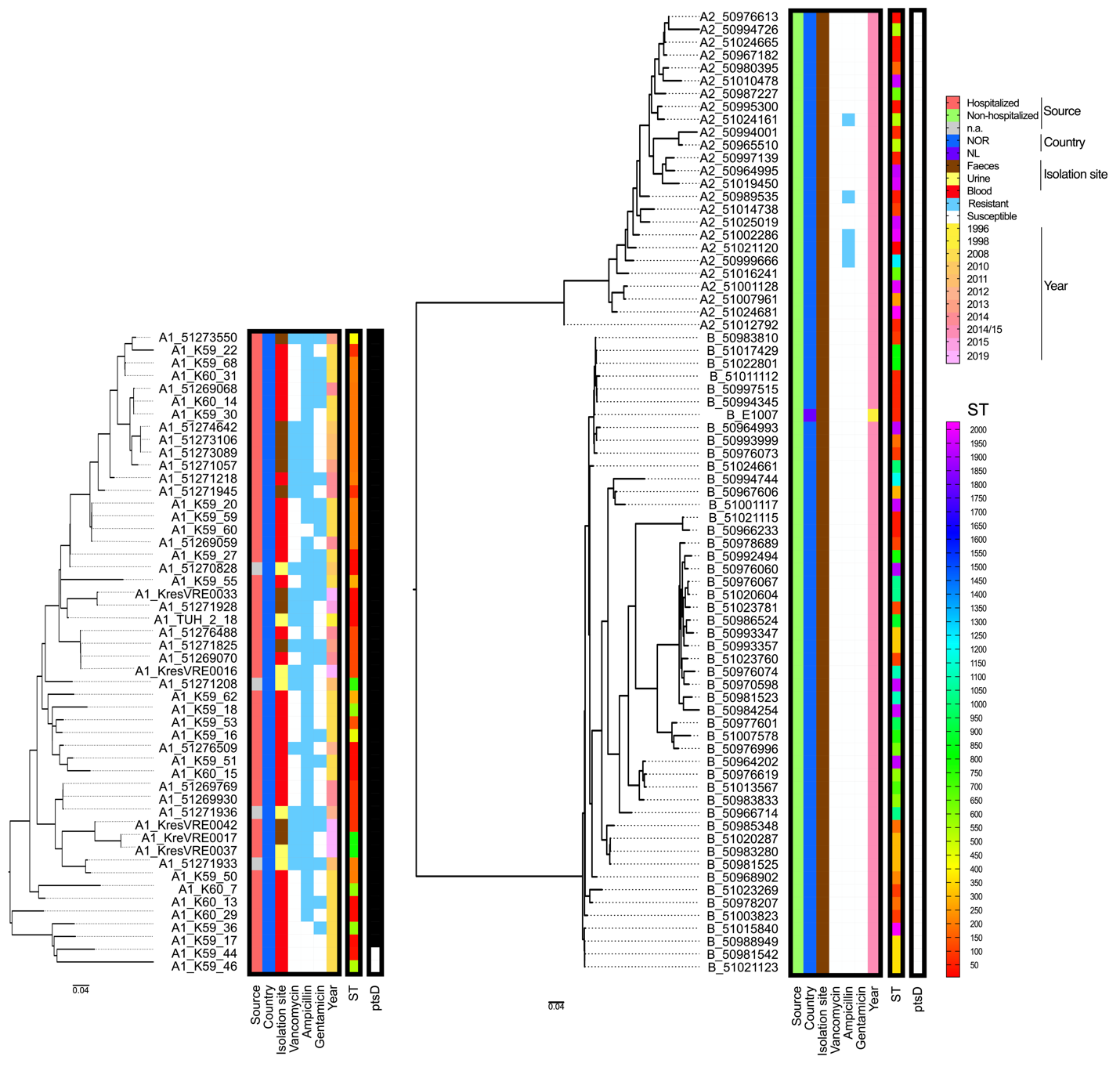


**Figure S5. *ptsD* in the strain collection.** The presence (black) or absence (white) of the *ptsD* gene in WGS data is indicated as a black bar.

**Figure S6. Growth curve of *E. faecium* E8202 and E8202 *ptsD*::GM.** a) Growth in BHI and BHI with 2 g/L mannose (BHI M), b) growth in LB and LB with 2 g/L mannose (LB M), c) growth in MH and MH with 2 g/L mannose (MH M), d) comparison of slopes of growth curves (one-way ANOVA). Biological triplicates and technical duplicates (n=6), median with SD.

**Figure S7. Interaction screening of different clades with *E. faecium* E8202 and E8202 *ptsD*::GM.** a) Clade A2 and B (*E. lactis*) strains (y-axis) as spots on *E. faecium* E8202 and E8202 *ptsD*::GM (x-axis) as target lawns. b) Clade A1 strains (y-axis) as spots on *E. faecium* E8202 and E8202 *ptsD*::GM (x-axis) as target lawns. The assay was conducted on different agar plates: BHI, BHI M (BHI with 2 g/L mannose), MH and LB. Inhibition is rated as indicated.
